# Legume Fingerprinting through Lipid Composition: Utilizing GC/MS with Multivariate Statistics

**DOI:** 10.3390/foods12244420

**Published:** 2023-12-09

**Authors:** Marko Ilić, Kristian Pastor, Aleksandra Ilić, Mirjana Vasić, Nataša Nastić, Đura Vujić, Marijana Ačanski

**Affiliations:** 1 Faculty of Technology Novi Sad, University of Novi Sad, 21000 Novi Sad, Serbia; kristian.pastor@uns.ac.rs (K.P.); natasa.nastic@uns.ac.rs (N.N.); macanski@tf.uns.ac.rs (M.A.); 2 Institute of Fields and Vegetable Crops, 21000 Novi Sad, Serbia; aleksandra.savic@ifvcns.ns.ac.rs (A.I.);; 3 Independent Researcher, 21000 Novi Sad, Serbia

**Keywords:** legumes, food authentication, GC/MS analysis, multivariate statistics, lipid profiles

## Abstract

This study presents a tentative analysis of the lipid composition of 47 legume samples, encompassing species such as *Phaseolus* spp., *Vicia* spp., *Pisum* spp., and *Lathyrus* spp. Lipid extraction and GC/MS (gas chromatography with mass spectrometric detection) analysis were conducted, followed by multivariate statistical methods for data interpretation. Hierarchical Cluster Analysis (HCA) revealed two major clusters, distinguishing beans and snap beans (*Phaseolus* spp.) from faba beans (*Vicia faba*), peas (*Pisum sativum*), and grass peas (*Lathyrus sativus*). Principal Component Analysis (PCA) yielded 2D and 3D score plots, effectively discriminating legume species. Linear Discriminant Analysis (LDA) achieved a 100% accurate classification of the training set and a 90% accuracy of the test set. The lipid-based fingerprinting elucidated compounds crucial for discrimination. Both PCA and LDA biplots highlighted squalene and fatty acid methyl esters (FAMEs) of 9,12,15-octadecatrienoic acid (C18:3) and 5,11,14,17-eicosatetraenoic acid (C20:4) as influential in the clustering of beans and snap beans. Unique compounds, including 13-docosenoic acid (C22:1) and γ-tocopherol, O-methyl-, characterized grass pea samples. Faba bean samples were discriminated by FAMEs of heneicosanoic acid (C21:0) and oxiraneoctanoic acid, 3-octyl- (C18-ox). However, C18-ox was also found in pea samples, but in significantly lower amounts. This research demonstrates the efficacy of lipid analysis coupled with multivariate statistics for accurate differentiation and classification of legumes, according to their botanical origins.

## 1. Introduction

Domestication of major legume crops occurred in parallel with that of cereals, at the very beginning of agriculture, making legumes one of the world’s oldest-grown crops [1,2]. When we are talking about systematics, it must be mentioned that *Fabaceae* (*Leguminosae*) is the third-largest family of flowering plants (800 genera and 20,000 species). According to morphological characters, three main subfamilies are recognized—*Mimosoideae*, *Papilionoideae* (*Faboideae*), and *Caesalpinoideae* [3]. All known cultivated crops belong to the subfamily *Papilionoideae*, which is further divided into several tribes. Only two tribes will be considered in this paper: (i) tribe Fabeae, including pea (*Pisum sativum* L.), faba bean (*Vicia faba* L.), and grass pea (Lathyrus *sativus* L.); and (ii) tribe Phaseoleae, including common and snap bean (*Phaseolus vulgaris* L.), scarlet runner bean (*Phaseolus coccineus* L.) and lima bean (*Phaseolus lunatus* L.), among other species.

Pea (*Pisum sativum* L.) belongs to the genus *Pisum* and is native to Europe (Mediterranean region) and Middle to Northwest Asia. Pea is among the most valuable temperate legumes, grown at high elevations or during cool seasons, used in the human diet, and also as fodder and green manure [4]. Pea can be used in the human diet as leaves, green pods, unripe and dry mature seed, while in animal feed it is used for direct grazing, hay, and silage. Pea also contributes to the reduction of Se (selenium) and folate deficiency-related diseases [5]. Faba bean (*Vicia faba* L.) belongs to the genus *Vicia*, with the Middle East stated as its center of origin [6]. In present times, Faba bean is widespread in Europe, North Africa, Central Asia, China, and the Americas [7]. It is usually consumed as a vegetable in the form of fresh or frozen immature seeds and pods, but it is also an important green-manure legume. In temperate regions where soybean cannot be grown, faba bean provides an alternative for animal feed [1]. A feature that distinguishes faba bean from other legumes is the production of L-DOPA (L-3.4-dihydroxyphenylalanine), a bioactive compound used for treating Parkinson’s disease [8]. Grass pea (*Lathyrus sativus* L.) and other species of the genus *Lathyrus* are native to the Mediterranean region (primary center of origin) and Central Asia (secondary center of origin). When, where, and which species of this genus were domesticated is not reliably established, but it is certain that it was known and used by the first hunters and gatherers from the Neolithic period [9]. Grass pea has the ability to survive extreme conditions (droughts, heat stress, flooding) showing good adaptability to various environments. It is often cultivated in developing countries. Even though grass pea provides benefits in the human diet, it can also produce a negative impact on health due to the presence of amino acid β-diaminopropionic acid (ODAP) with proven neurotoxic effects. This neurotoxin causes a disease called lathyrism, an irreversible neurological disorder that can permanently paralyze adults from the knee down and cause brain damage in children [10]. However, this only happens when consumed in larger quantities, usually during periods of famine.

Common bean (*Phaseolus vulgaris* L.) is the second most important food legume, just after the soybean. Its wide distribution is explained by good adaptability to diverse agroecological conditions and production systems, as well as its variety in colors, shapes, sizes, and types of use (dry and fresh seeds, fresh pods, etc.) [11]. In the developing world, common and snap beans are grown for home and local consumption, whereas in the developed countries, the focus is placed on tprocessing and export. Common bean, together with several other crop relatives (tepary beans—*Phaseolus acutifolius*; scarlet runner bean—*Phaseolus lunatus*; year-long bean—*Phaseolus dumosus*) belong to the genus *Phaseolus*. Even though all these species have the same number of chromosomes, their mutual breeding comes with difficulties, leading to the organization of these species into secondary, tertiary, and quaternary gene pools in relation to common bean [12]. Therefore, *Phaseolus coccineus* belongs to the secondary gene pool with possible delivery of introgression lines with common bean, while *Phaseolus lunatus* falls into the quaternary gene pool of common bean without confirmed crosses between these two species. Domestication of *Phaseolus* genus took place in Central and South America with different dissemination pathways for each species [13]. Common bean quickly spread and is widely grown in Africa, Asia, and Europe, while scarlet runner bean is locally cultivated in parts of the Americas, Asia, and Africa, but can be found exported in European markets. On the other hand, the lima bean is considered a food security crop in parts of the Caribbean, Mexico, and Peru [1].

Authentication analyses of plant ingredients are essential for bolstering consumer confidence and combating instances of false labeling and adulteration. The Grocery Manufacturers Association of America has reported that food fraud affects up to 10% of food in the developed world and 20% in the developing world [14]. For instance, in legume markets, different species, especially those with high value due to their origin, are sold under the same name and, therefore, there is a great need for developing accurate methods of proving the authenticity of raw materials [15].

There are many examples of the application of a GC/MS technique (gas chromatography with mass spectrometric detection) coupled with various multivariate data analysis approaches for the purpose of food authentication and fraud detection. Thus, lipid profiles showed to be a useful input in: the authentication of the botanical origin of various types of edible oils [16,17,18,19]; classification of cereal flours [20], cereal and pseudocereal flours [21], and common and sourdough bread [22]; Parmigiano Reggiano authentication [23]; differentiation between organically raised lamb and broiler compared to conventionally raised meats [24,25]; characterization of different mushroom species [26]; and many other applications.

Caprioli et al. [27] investigated the lipid nutritional value of particular legume samples in terms of fatty acid composition, showing that some types of legumes contain interesting values for the ratio of polyunsaturated fatty acids (PUFAs) *n*-6/*n*-3, even reaching 57.5%. The highest detected level of saturated acids was 37.4%, with palmitic acid being higher than stearic in all samples. The levels of monounsaturated oleic acid varied vastly, from 5.1 to 39.6%. The aim of this paper was to develop a legume authentication method based on lipid composition. Analysis and identification of extracted lipid components were processed by the GC/MS technique. Multivariate statistic tools were used for the differentiation and classification of the legume samples.

## 2. Materials and Methods

### 2.1. Sample Composition

Legume samples were collected from the Institute of Field and Vegetable Crops, Novi Sad, Serbia. Number of legume samples was 47 in total: 15 beans (samples 1–9 and 14–15 *Phaseolus vulgaris*, sample 16 *Phaseolus lunatus*, and samples 17–19 *Phaseolus coocineus*), 4 snap beans (samples 10–13 *Phaseolus vulgaris*), 8 faba beans (samples 20–27 *Vicia faba)*, 7 peas (samples 28–34 *Pisum sativum*), and 13 grass peas (samples 35–47 *Lathyrus sativus*).

### 2.2. Lipid Extraction

All legume samples were dried in the air and ground into flour. The mass of 0.5 g of each sample was measured on an analytical balance with a precision of 0.0001 g. The volume of 2 mL of *n*-hexane was added, then vortexed for 10 s and centrifuged at 3000 rpm for 7 min. The supernatant (hexane fraction) was decanted and the residual hexane evaporated overnight. Reconstitution was performed by adding 1 mL of dichloromethane (DCM), followed by trans-derivatization using a 0.2 M trimethylsulfonium hydroxide (TMSH) solution in methanol. Trans-derivatization was performed in the injection port of the gas chromatography device [20].

### 2.3. GC/MS Analysis

The analysis was performed using a gas chromatography device (Agilent Technologies 7890) coupled to a mass spectrometric detector (Agilent Technologies 5975 MSD). Electron ionization was applied, with an energy of 70 eV. Helium (purity of 5.0 helium = 99.999% purity) was employed as a carrier gas through the system with a flow of 1 mL min^−1^. A DB-5 MS column (Agilent Technologies, 30 m × 0.25 mm × 0.25 μm) was used to separate the eluting molecules. The temperature of the injection port was 250 °C, and the applied temperature program was 50–130 °C at 30 °C min^−1^ and 130–280 °C at 15 °C min^−1^ [19]. A volume of 1 μL of each sample was injected with a split ratio of 10:1. The mass range of 45–500 *m*/*z* was scanned.

### 2.4. Data Acquisition

An offline ChemStation B.04.03 software (Agilent Technologies, Palo Alto, CA, USA) was employed to process the obtained chromatograms. The mass spectra of the peaks were compared to the NIST mass spectra library to tentatively identify the eluting compounds. Only compounds with over 80% matches were considered identified. Peak areas of all identified compounds were used to create the data matrix for further data processing. 

### 2.5. Data Analysis

Collected peak areas of each identified compound were employed as variables in multivariate data processing. The values were divided by the highest among them to normalize the data in the range between 0 and 1. Multivariate statistics was employed for further analysis of the obtained dataset. Multivariate statistic tools: Hierarchical cluster analysis (HCA), Principal component analysis (PCA), and Linear discriminant analysis (LDA) were applied using PAST 4.0 software (National History Museum, University of Oslo, Norway). As unsupervised tools, both HCA and PCA focus on the overall structure or similarity of the data, while LDA, as a supervised tool, considers class information and aims to find the linear combinations of features that best separate different classes. In order to improve the reliability and the quality of the representation of the applied LDA classification model the analyzed normalized data were separated into a training set (37 samples, or 79%) and a test set (10 samples or 21%), and the accuracies of both sets were calculated. The data PCA and LDA biplots were employed to fingerprint analyze legume samples based on obtained lipid profiles.

## 3. Results

### 3.1. Data Acquisition

Lipid extracts obtained from the legume samples were analyzed by GC/MS device, followed by a tentative identification of the eluting compounds, which are presented in Table 1. Abbreviations, retention times, and match ranges of the compounds are also given. Matches of the compounds identified in all legume samples were over 80%. Typical examples of the chromatograms obtained by GC/MS analysis are presented in Figures S1–S5 (Supplementary Files).

### 3.2. Hierarchical Cluster Analysis

**Figure 1:**
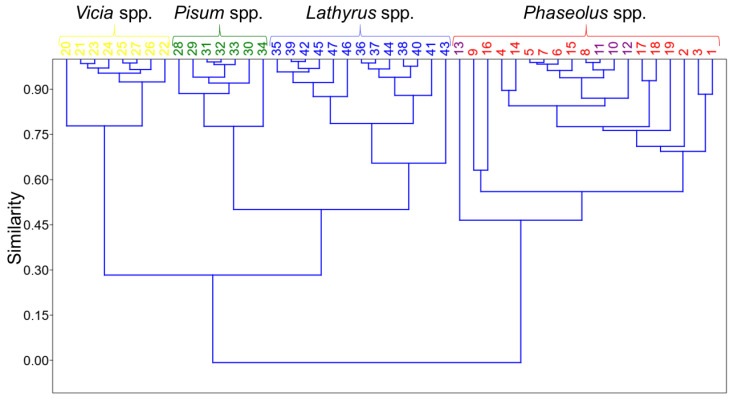
Similarity dendrogram of the legume samples clustering based on lipid composition.

Hierarchical cluster analysis (HCA) was performed on the data matrix using the *Paired group* algorithm and *Rho* similarity index. A similarity dendrogram with two major clusters was obtained and presented in Figure 1. One major cluster with the similarity at the level of about 0.45, contains bean and snap bean samples. The presence of these two groups of samples in the same cluster was expected due to their belonging to the same tribe Phaseoleae, and also the same genus *Phaseolus* including three species (*Phaseolus vulgaris*, *Phaseolus lunatus*, and *Phaseolus coocineus*). In fact, beans and snap beans are essentially different stages of the same plant species. 

Samples of faba bean (*Vicia faba*), pea (*Pisum sativum*), and grass pea (*Lathyrus sativus*) are clearly separated within the other major cluster with a similarity at the level of about 0.30. These three legume species belong to the same tribe Fabeae, and are therefore discriminated from the *Phaseolus* spp. cluster. Similarities of each faba bean and pea cluster are both at the level of about 0.75, while the grass pea cluster shows the similarity at the level of about 0.60. 

### 3.3. Principal Component Analysis

In this study, the utilization of both HCA and PCA as unsupervised pattern recognition methods serves specific and complementary purposes in visualizing the data. In essence, the integration of both unsupervised tools in this study enhances the depth and breadth of data exploration. HCA delves into the intricate relationships between samples, while PCA distills the essential features driving variability. This combined approach provides a more holistic understanding of the underlying patterns in the dataset, offering a richer foundation for subsequent analyses and interpretations. 

**Figure 2:**
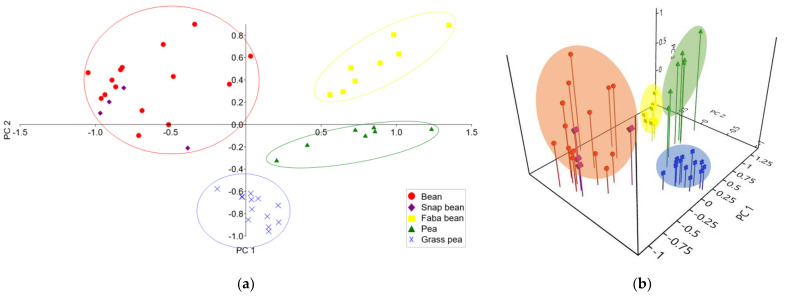
Lipid composition-based discrimination of the legume samples by PCA: (**a**) 2D score plot; (**b**) 3D score plot.

Principal component analysis (PCA) was employed to create both 2D and 3D score plots, based on the collected data. First, three principal components (PCs) accounted for 54.86%, 24.73%, and 16.88% of the total variance, respectively. In Figure 2a, a 2D PCA score plot is presented as PC 1 versus PC 2, accounting for almost 80% of the total variance. Four clusters were obtained, discriminating legume samples properly, according to their botanical origin. Faba bean samples are clustered in the first quadrant of the PCA score plot. Bean and snap bean samples form a common cluster mostly in the second quadrant, similar to the dendrogram in Figure 1. The grass pea samples cluster is located on the border of the third and fourth quadrants, but mostly in the fourth. Pea samples are also located in the fourth quadrant but are clearly separated from grass pea samples.

Figure 2b shows a 3D PCA score plot as PC 1 versus PC 2 versus PC 3, accounting for over 96% of the total variance. By including the third principal component, a higher level of sample differentiation was obtained. Namely, pea and grass pea samples are both located in the fourth quadrant in the 2D PCA score plot. However, in the 3D PCA score plot, these samples are discriminated by PC 3. Pea samples are located in the fourth octant, while grass pea samples are mostly located in the eighth octant. Bean and snap bean samples are clustered together in the second and sixth octants. The faba bean cluster is located in the fifth octant.

### 3.4. Linear Discriminant Analysis

On the same data matrix, linear discriminant analysis (LDA) was performed. As already mentioned, the samples were separated into a training set and a test set. The training set includes 37 samples (79% of the data), while the test set includes 10 randomly chosen samples (21% of the data), at least one of each analyzed legume species.

**Figure 3:**
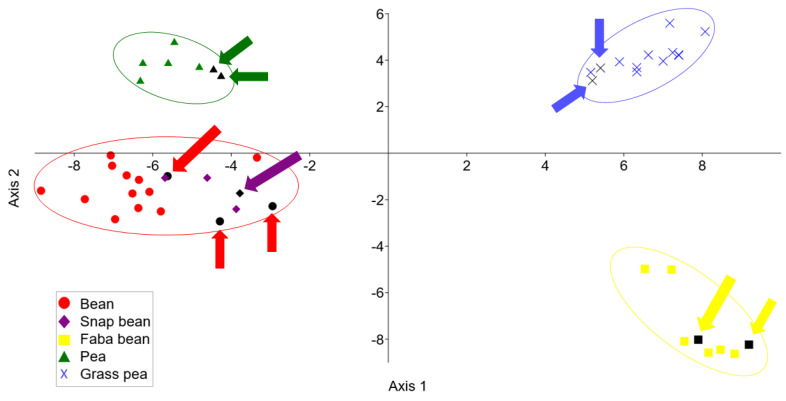
LDA score plot of the lipid-based legume discrimination.

The obtained LDA score plot is shown in Figure 3. Legume samples are clustered according to their species. Clusters took place in four different quadrants: grass pea in the first quadrant, pea in the second, bean and snap bean in the third, and faba bean in the fourth quadrant. As in the case of HCA and PCA, bean and snap bean samples are also clustered together by LDA due to their belonging to the same tribe Phaseoleae and the same genus *Phaseolus*. The test set samples are colored in black and specifically marked. These samples are clustered with the other samples of their own species.

LDA was also employed to classify the samples due to their botanical origin. The confusion matrix based on the training set of analyzed legume samples is shown in Table 2. Each row in the matrix presents a given group, while each column presents a predicted group. In the case of the training set, LDA resulted in 100% correctly classified samples. Table 3 presents the obtained confusion matrix based on all analyzed samples including the test set. Out of 10 samples of the test set, 9 samples are correctly classified indicating 90% of model accuracy. Only 1 sample is classified incorrectly. The misclassified bean sample belongs to the predicted group of snap beans. This could be expected since the LDA score plot has not distinguished bean and snap bean samples.

### 3.5. Fingerprinting

**Figure 4:**
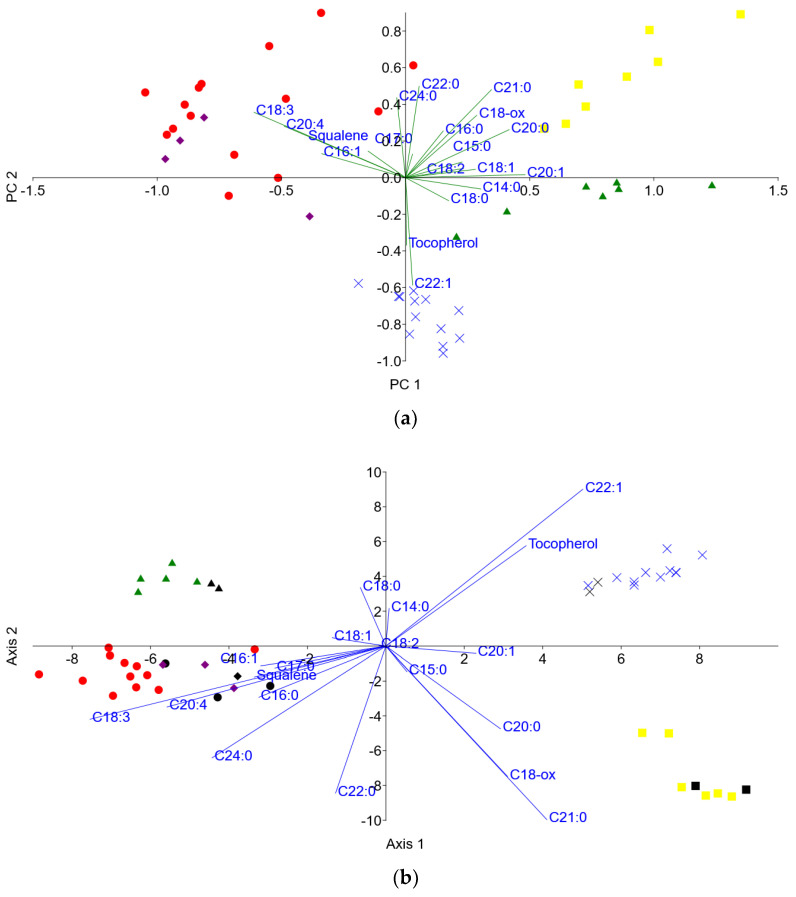
(**a**) PCA biplot and (**b**) LDA biplot of the lipid components influence on the legume discrimination.

Both PCA and LDA biplots (Figure 4a,b, respectively) are employed in lipid-based legume fingerprinting. Both biplots distinguish 9,12,15-octadecatrienoic acid, methyl ester (C18:3, linolenic acid) and 5,11,14,17-eicosatetraenoic acid, methyl ester (C20:4, arachidonic acid) as the fatty acid methyl esters (FAMEs) with the most dominant influence on the clustering of bean and snap bean samples. According to the PCA biplot, squalene also shows a significant influence on the differentiation of this cluster. Of all analyzed legume samples, these three lipid compounds were found only in bean and snap bean samples.

It was found that 13-docosenoic acid, methyl ester (C22:1, erucic acid), and γ-tocopherol, O-methyl- are present only in grass pea samples, which is confirmed both by PCA and LDA biplots. 

According to both PCA and LDA biplots, eicosanoic acid, methyl ester (C20:0, arachidic acid), heneicosanoic acid, methyl ester (C21:0), and oxiraneoctanoic acid, 3-octyl-, methyl ester (C18-ox) show the most significant influence on clustering of faba bean samples. C21:0 was found only in faba bean samples; however, this is not the case for the other two compounds. Besides faba bean, C18-ox was also found in pea samples but in significantly lower values of peak surface areas. On the contrary, C20:0 was found in all analyzed legume samples, so it can’t be considered for fingerprinting.

## 4. Discussion

Los et al. [28] and Chaurasia [29] showed that *Phaseolus vulgaris* varieties have a high proportion of unsaturated fatty acids, with linolenic (C18:3n3) and linoleic (C18:2n6) acids as the main fatty acids (62–83% of total fatty acids). The beans’ abundant omega-3 polyunsaturated fatty acids (PUFA) contribute to a favorable omega-6/omega-3 ratio, potentially yielding health benefits. They are essential components of cell membranes and contribute to various physiological processes associated with cardiovascular health, reducing inflammation, and supporting cognitive function. They may also play a role in the prevention of chronic diseases, improving insulin sensitivity, and lowering the incidence of type 2 diabetes and anti-arrhythmic effects. On the contrary, high levels of erucic acid have been linked to adverse effects on heart health in animal studies. It is also important to note that while some saturated fats are necessary for health, excessive intake of saturated fats, such as Arachidonic acid, particularly those from unhealthy sources, has been associated with certain health risks, including cardiovascular issues [27]. 

Lipid-based fingerprinting was revealed by PCA and LDA biplots presented in Figure 4. Although they both give the same conclusions, there are some differences that can be pointed out. The LDA biplot clearly indicates potential biomarkers in the case of faba beans. On the other hand, in the cases of beans and snap beans, the PCA biplot has been shown to be a better choice for indicating potential biomarkers. There are no lipid biomarkers found in peas, which is confirmed by the LDA biplot. Both biplots are shown to be equally useful tools in the case of revealing grass pea lipid biomarkers.

Huschek et al. [14] presented a strategy to identify novel biomarkers for a targeted proteomics LC-MS/MS that can simultaneously prove the presence/absence of garden pea, a protein-rich legume, meat, and honey and quantify their content in processed vegan food. LC/MS-based untargeted metabolomics was also applied to discriminate between three legume species: *Cicer arietinum* L. (chickpea), *Lens culinaris* L. (lentil), and *Phaseolus vulgaris* L. (white bean) [30]. The authors identified 43 compounds, among which polyphenols, fatty acyls, prenol lipids, a nucleoside, and organic compounds, qualified as discriminating variables. Furthermore, cowpea (*Vigna unguiculata*), pigeon pea (*Cajanus cajan*), and common bean (*Phaseolus vulgaris*) were discriminated using two distinct UPLC-MS platforms and analytical workflows coupled to HCA, PCA, and one-way ANOVA [31]. Lentil samples were geographically authenticated using ^1^H NMR [32] and ICP-OES [33] coupled to PCA and LDA chemometric tools. Elemental profiles established with F-AAS were also employed to classify legume and oilseed samples available in the Polish market in view of their botanical species and type [34]. However, by applying Factor and Cluster analyses, the authors did not manage to fully discriminate the investigated legumes. 

On the other hand, a completely different methodology employing DNA analysis was proposed by Madesis et al. [15]. A highly flexible and coupled real-time PCR and HRM analysis approach was found to be effective for authenticating bean species, being able to detect admixtures and adulterants in food products in the field of DNA detection in foods. Lioi et al. [35] and Omar et al. [36] employed gel electrophoresis to separate proteins extracted from the legume samples. They performed PCR amplification furthered by phylogenetic analysis to discriminate the analyzed legume species. Yildizdogan et al. [37] performed amplified fragment length polymorphism (AFLP) on the extracted DNA samples coupled with PCA (2D and 3D) to differentiate *Cicer* L., *Lathyrus* L., *Lens* Mill., and *Vicia* L. samples. However, differentiation has not been completely achieved. 

According to the authors’ knowledge, this is the first application of lipid profiles established using a GC/MS system with the aim of differentiating and authenticating legume species according to their botanical origin. Thus, this is a new approach showing great potential in legume authentication. Although significant and meaningful results were obtained in the presented study, some future explorations on a larger sample amount and other legume genera and species need to be conducted in order to achieve more reliable conclusions and routinely apply the proposed methodology.

## 5. Conclusions

In conclusion, this study presents a robust approach for the differentiation and classification of various legume species based on their lipid composition. The combination of GC/MS analysis and multivariate statistical methods, including Hierarchical Cluster Analysis (HCA), Principal Component Analysis (PCA), and Linear Discriminant Analysis (LDA), proved to be highly effective. HCA revealed distinct clusters, emphasizing the botanical relationships among legumes, while PCA provided insightful 2D and 3D score plots, visually representing the separation of different legume species. LDA further refined the classification, achieving a 100% accuracy of the training set and 90% accuracy of the test set in predicting the botanical origin of samples by the obtained model. The lipid-based fingerprinting facilitated the identification of key compounds influencing the differentiation of legumes. This research contributes not only to the understanding of the lipid profiles of different legumes but also highlights the potential for lipid analysis as a powerful tool for botanical classification. The comprehensive insights gained from this study enhance our ability to distinguish legume species accurately, offering valuable implications for fields such as agriculture, nutrition, and food science.

## Figures and Tables

**Table 1:** Identified lipid compounds extracted from the legume samples, with their abbreviations and retention times.

Compound	Abbr. *	Retention Time [min]	Match Range [%]
Tetradecanoic acid, methyl ester	C14:0	8.74	81.2–95.2
Pentadecanoic acid, methyl ester	C15:0	9.44	80.7–92.2
9-Hexadecenoic acid, methyl ester	C16:1	10.00	80.0–89.9
Hexadecanoic acid, methyl ester	C16:0	10.13	92.5–97.3
Heptadecanoic acid, methyl ester	C17:0	10.79	80.1–86.5
9,12-Octadecadienoic acid, methyl ester	C18:2	11.25	89.6–96.7
9,12,15-Octadecatrienoic acid, methyl ester	C18:3	11.29	82.5–95.6
9-Octadecenoic acid, methyl ester	C18:1	11.30	80.0–93.6
Stearic acid, methyl ester	C18:0	11.42	91.4–95.9
5,11,14,17-Eicosatetraenoic acid, methyl ester	C20:4	12.36	81.8–84.4
Oxiraneoctanoic acid, 3-octyl-, methyl ester	C18-ox	12.44	81.9–92.6
11-Eicosenoic acid, methyl ester	C20:1	12.48	80.6–90.2
Eicosanoic acid, methyl ester	C20:0	12.61	81.7–93.0
Heneicosanoic acid, methyl ester	C21:0	13.20	80.5–83.4
13-Docosenoic acid, methyl ester	C22:1	13.71	80.7–87.9
Docosanoic acid, methyl ester	C22:0	13.86	80.1–90.5
Tetracosanoic acid, methyl ester	C24:0	15.50	80.2–88.2
Squalene	Squalene	16.41	81.0–91.7
γ-Tocopherol, O-methyl-	Tocopherol	18.40	87.0–90.9

* Abbr.—Abbreviation.

**Table 2:** Confusion matrix obtained by linear discriminant analysis of the training set of legume samples.

Groups *	Bean	Snap Bean	Faba Bean	Pea	Grass Pea	Total
Bean	12	0	0	0	0	12
Snap bean	0	3	0	0	0	3
Faba bean	0	0	6	0	0	6
Pea	0	0	0	5	0	5
Grass Pea	0	0	0	0	11	11
Total	12	3	6	5	11	37

* Rows—given groups, columns—predicted groups.

**Table 3:** Confusion matrix obtained by linear discriminant analysis of legume samples based on lipid composition.

Groups *	Bean	Snap Bean	Faba Bean	Pea	Grass Pea	Total
Bean	14	1	0	0	0	15
Snap bean	0	4	0	0	0	4
Faba bean	0	0	8	0	0	8
Pea	0	0	0	7	0	7
Grass Pea	0	0	0	0	13	13
Total	14	5	8	7	13	47

* Rows—given groups, columns—predicted groups.

## Data Availability

All data generated or analyzed during this study are available from the corresponding author upon reasonable request.

## Supplementary Materials

The following supporting information can be downloaded at: https://www.mdpi.com/article/10.3390/foods12244420/s1, Figure S1: An example of the chromatogram obtained by GC/MS analysis of bean samples; Figure S2: An example of the chromatogram obtained by GC/MS analysis of snap bean samples; Figure S3: An example of the chromatogram obtained by GC/MS analysis of faba bean samples; Figure S4: An example of the chromatogram obtained by GC/MS analysis of pea samples; Figure S5: An example of the chromatogram obtained by GC/MS analysis of grass pea samples.
