# Supplementary material for: Legume Fingerprinting through Lipid Composition: Utilizing GC/MS with Multivariate Statistics
Source: Foods. 2023 Dec 9;12(24):4420. doi: 10.3390/foods12244420 (PMC10742467; doi:10.3390/foods12244420)
Supplement: Supplementary file 1 [file foods-12-04420-s001.zip › foods-2735659-supplementary.pdf]

Supplementary Files

Legume Fingerprinting through Lipid Composition: Utilizing GC/MS with Multivariate Statistics

Marko Ilić [1,*], Kristian Pastor [1], Aleksandra Ilić [2], Mirjana Vasić [2], Nataša Nastić [1], Đura Vujić [3] and Marijana Ačanski [1]

[1] Faculty of Technology Novi Sad, University of Novi Sad, 21000 Novi Sad, Serbia; kristian.pastor@uns.ac.rs (K.P.); natasa.nastic@uns.ac.rs (N.N.); macanski@tf.uns.ac.rs (M.A.)
[2] Institute of Fields and Vegetable Crops, 21000 Novi Sad, Serbia; aleksandra.savic@ifvcns.ns.ac.rs (A.I.)
[3] Independent Researcher, 21000 Novi Sad, Serbia
* Correspondence: marko.ilic@uns.ac.rs; Tel.: +381-21-485-3692

Typical examples of the chromatograms obtained by GC/MS analysis of legume samples, are presented in Figures S1–S5.

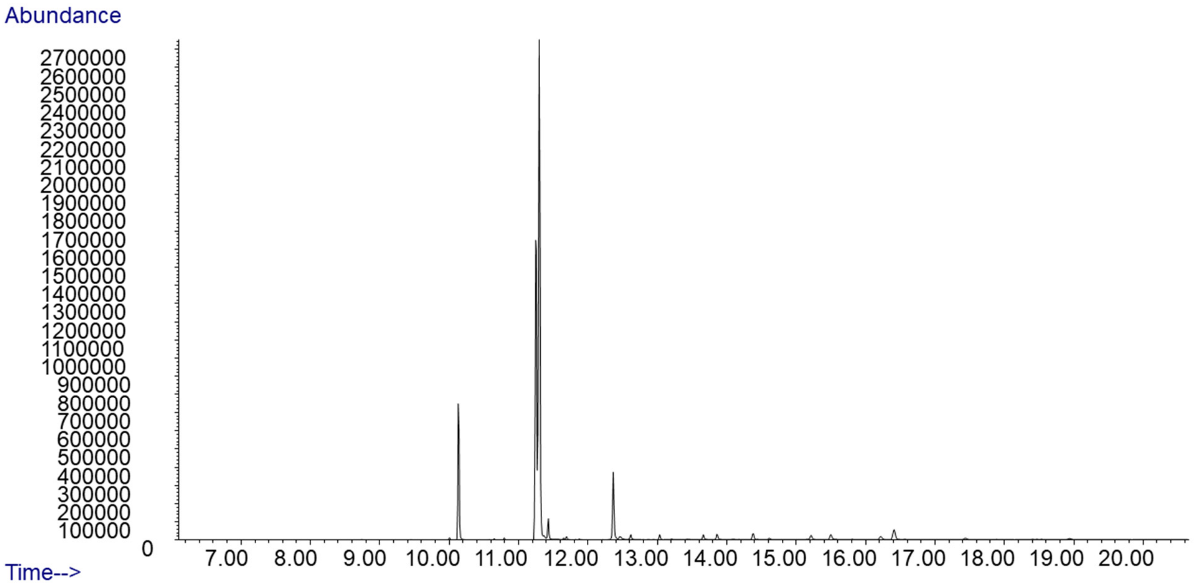

Figure S1. An example of the chromatogram obtained by GC/MS analysis of bean samples.

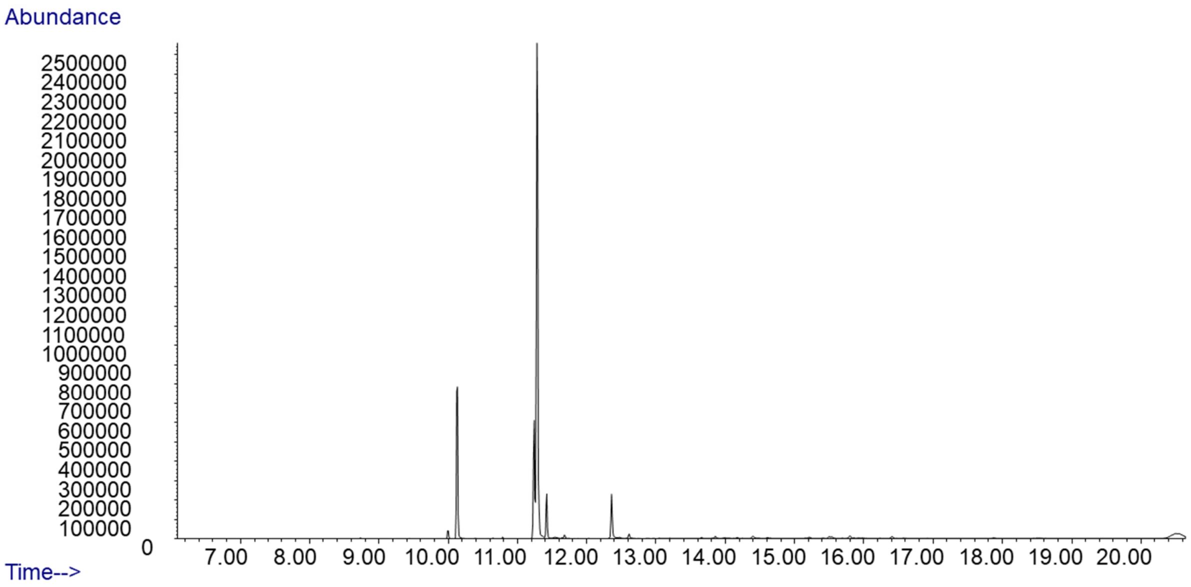

Figure S2. An example of the chromatogram obtained by GC/MS analysis of snap bean samples.

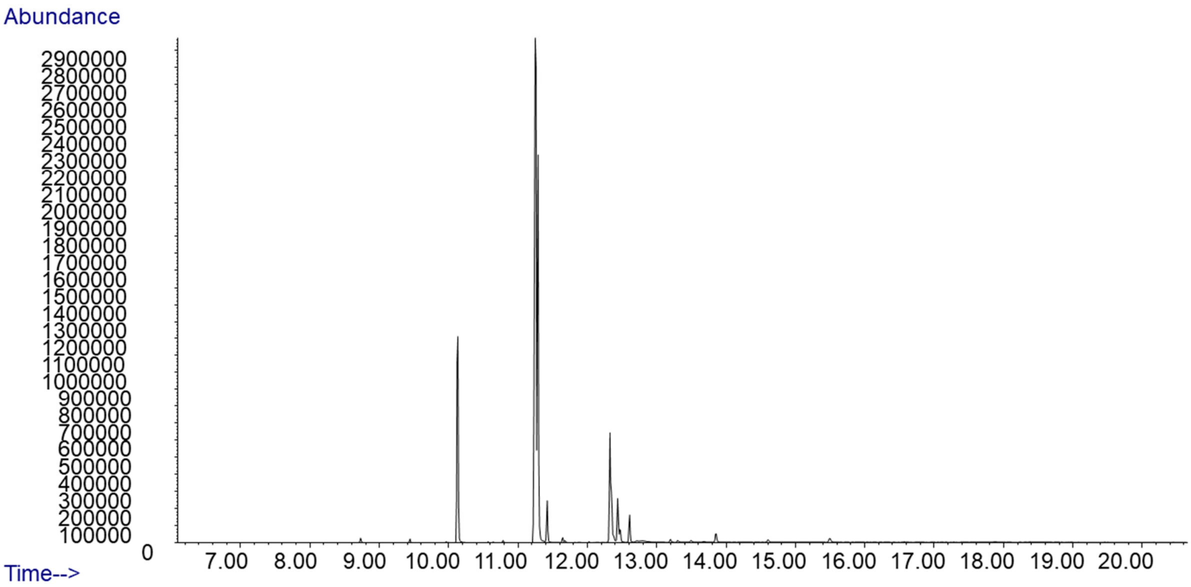

Figure S3. An example of the chromatogram obtained by GC/MS analysis of faba bean samples.

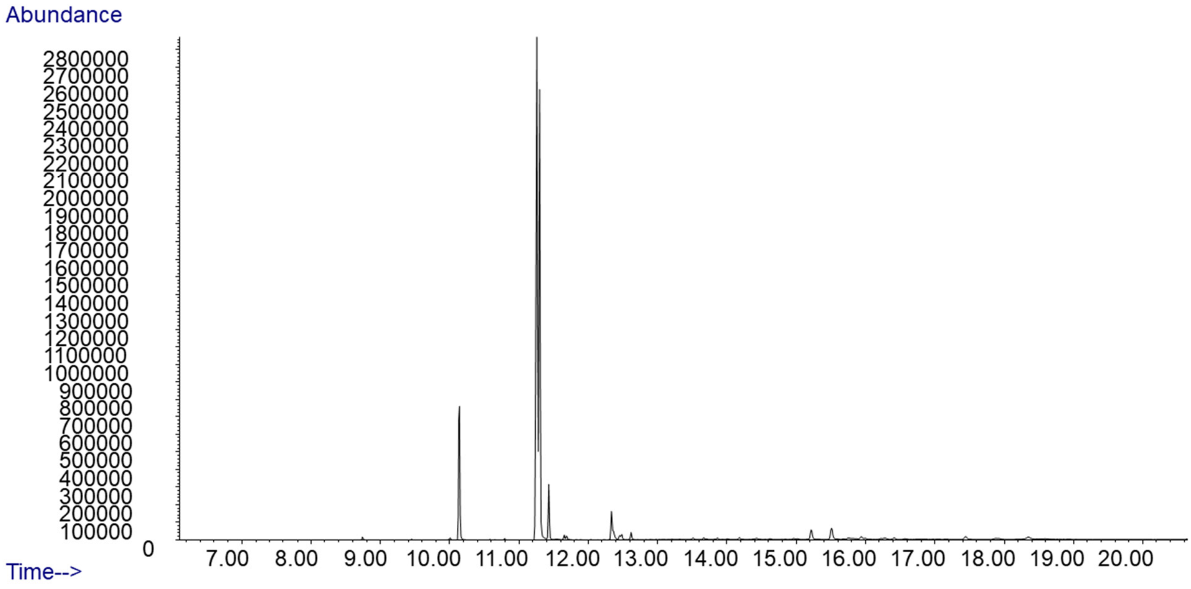

Figure S4. An example of the chromatogram obtained by GC/MS analysis of pea samples.

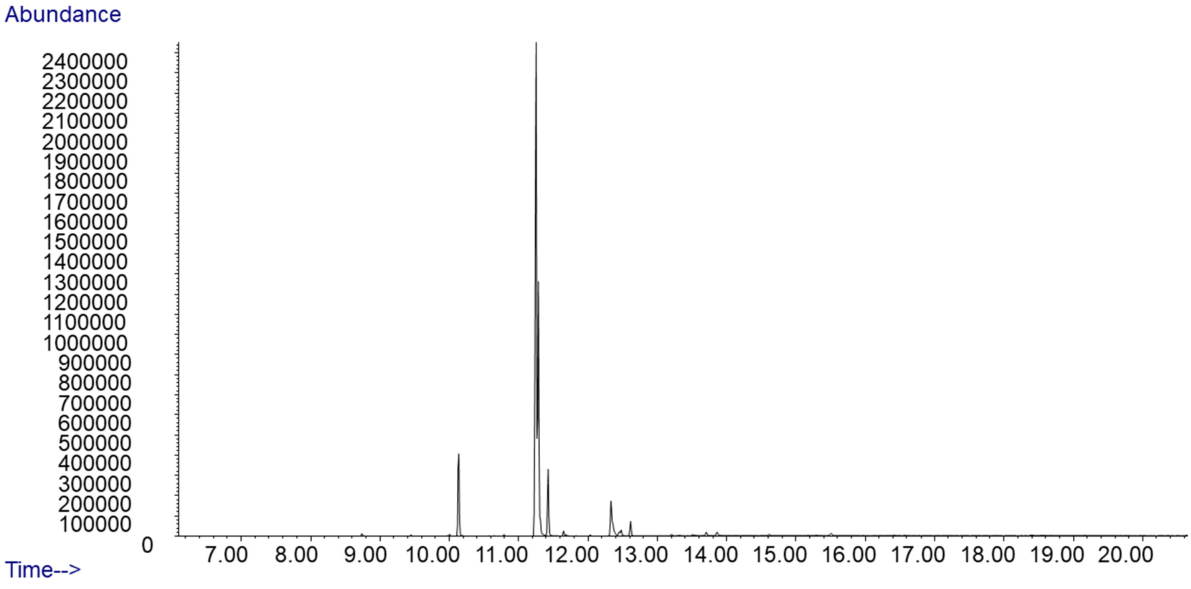

Figure S5. An example of the chromatogram obtained by GC/MS analysis of grass pea samples.
